# Excellent Infrared Nonlinear Optical Crystals BaMO(IO_3_)_5_ (M = V, Ta) Predicted by First Principle Calculations

**DOI:** 10.3390/ma11101809

**Published:** 2018-09-24

**Authors:** Yingfeng Li, Mengqi Cui, Hejin Yan, Yangxin Yu, Meicheng Li, Xiang Li, Lihua Chu, Bing Jiang, Mingde Qin

**Affiliations:** 1 State Key Laboratory of Alternate Electrical Power System with Renewable Energy Sources, School of Renewable Energy, North China Electric Power University, Beijing 102206, China; cmqroy@126.com (M.C.); li-bluesky@hotmail.com (H.Y.); mcli@ncepu.edu.cn (M.L.); 13051314181@163.com (X.L.); 51102229@ncepu.edu.cn (L.C.); mucaoshan@163.com (B.J.); 2 Laboratory of Chemical Engineering Thermodynamics, Department of Chemical Engineering, Tsinghua University, Beijing 100084, China; 3 State Key Laboratory of Chemical Engineering, Department of Chemical Engineering, Tsinghua University, Beijing 100084, China; 4 Program of Materials Science and Engineering, University of California, San Diego, CA 92093, USA; mingdeqin2@gmail.com

**Keywords:** SOJT, second harmonic generation, laser damage threshold, density functional theory

## Abstract

Two nonlinear optical crystals, BaVO(IO_3_)_5_ and BaTaO(IO_3_)_5_, are designed by substituting Nb with V and Ta, respectively, in BaNbO(IO_3_)_5_, which is itself a recently synthesized infrared nonlinear optical (NLO) material. The designs of BaVO(IO_3_)_5_ and BaTaO(IO_3_)_5_ from BaNbO(IO_3_)_5_ are based on the following motivation: BaVO(IO_3_)_5_ should have a larger second-harmonic generation (SHG) coefficient than BaNbO(IO_3_)_5_, as V will result in a stronger second-order Jahn-Teller effect than Nb due to its smaller ion radius; at the same time, BaTaO(IO_3_)_5_ should have a larger laser-damage threshold, due to the fact that Ta has a smaller electronegativity leading to a greater band-gap. Established on reliable first-principle calculations, it is demonstrated that BaVO(IO_3_)_5_ has a much larger SHG coefficient than BaNbO(IO_3_)_5_ (23.42 × 10^−9^ vs. 18.66 × 10^−9^ esu); and BaTaO(IO_3_)_5_ has a significantly greater band-gap than BaNbO(IO_3_)_5_ (4.20 vs. 3.55 eV). Meanwhile, the absorption spectra and birefringences of both BaVO(IO_3_)_5_ and BaTaO(IO_3_)_5_ are acceptable for practice, suggesting that these two crystals can both be expected to be excellent infrared NLO materials.

## 1. Introduction

Great effort has been made in searching for new second-order nonlinear optical (NLO) materials for their increasingly important role in many advanced scientific and technological areas, especially in laser technology [1,2,3,4,5,6,7]. Among them, metal iodates attract a lot of attention as several them have shown large second-harmonic generation (SHG) coefficients, high laser-damage thresholds (LDT), wide transparent wavelength regions, and good thermal stability [8,9,10,11].

Recently, one metal iodate, BaNbO(IO_3_)_5_, was synthesized by Sun, et al. [12]. Its SHG coefficient reaches 14 times as that of KH_2_PO_4_(KDP); meanwhile, its band-gap (*E_g_*) is greater than 3.5 eV. It is well known that SHG and LDT (proportional to *E_g_*) are the two main indices for a NLO material, and the general criteria for the scientific research and industrial application of a NLO crystal are that the SHG coefficient should be 10 times larger than that of KDP (1.1 × 10^−9^ esu) [9], and *E_g_* should be greater than 3.0 eV. The SHG coefficient and *E_g_* determine that BaNbO(IO_3_)_5_ is a very promising NLO crystal that can be used in the infrared region. As the NLO crystals used in the infrared region are inadequate for applications at present [13,14,15], BaNbO(IO_3_)_5_ has been widely studied [16,17] since it was first synthesized.

According to the anionic group theory proposed by Chen [18], the SHG coefficients are mainly determined by the second-order Jahn-Teller (SOJT) effect of the anionic groups. In BaNbO(IO_3_)_5_, the anionic group NbO(IO_3_)_5_^2−^ is a typical non-centrosymmetric octahedron structure: Nb atom locates at the center of NbO_6_ octahedron and it links with five IO^3−^ and one terminal oxygen atom. The SOJT effect of NbO(IO_3_)_5_^2−^ comes from both the IO^3−^ group and the cation Nb^5+^ [19,20,21,22,23]. As the SHG response is in proportion to the SOJT effect, it is a natural idea to improve the SHG performance of BaMO(IO_3_)_5_ by enlarging the SOJT effect of its anionic group MO(IO_3_)_5_^2−^. The simplest way is to substitute the d^0^ transition metal Nb with other ones in the same family which can result in lager SOJT effect. It has been determined that the SOJT effect resulting from V, Nb and Ta follows the order V^5+^ > Nb^5+^ > Ta^5+^ [8]; therefore, it can be expected that BaVO(IO_3_)_5_ has a lager SHG efficient than BaNbO(IO_3_)_5_. In fact, a similar material Ba_2_VO_2_(IO_3_)_4_(IO_3_) [24] reported previously really shows larger out-of-center distortion, 1.16, than that of BaNbO(IO_3_)_5_, 0.63 [12]; the SHG coefficient of another similar crystal NaVO_2_(IO_3_)_2_(H_2_O) [25] synthesized by Yang et al. can reach 20 × KDP. Nevertheless, BaTaO(IO_3_)_5_ can be expected to have a greater *E_g_* than BaNbO(IO_3_)_5_ because the electronegativity decreases from V to Nb then to Ta. Therefore, BaVO(IO_3_)_5_ and BaTaO(IO_3_)_5_ should both be promising crystals for practical applications.

Based on the above idea, in this work, two nonlinear optical crystals BaMO(IO_3_)_5_ (M = V, Ta) have been proposed, and their NLO properties were predicted based on first-principles calculations. As there are still no reports on their syntheses, vibration analyses were firstly carried out to demonstrate their kinetic stability. To validate the thermodynamic stability of BaMO(IO_3_)_5_ (M = V, Ta), the ab initio molecular dynamics simulations were also performed. Then, to verify the precision and reliability of our prediction, comparisons between our calculated and the experimentally measured properties of BaNbO(IO_3_)_5_ were carried out. Finally, the SHG coefficient, *E_g_*, the birefringence Δ*n*, and the absorption spectrum of BaMO(IO_3_)_5_ (M = V and Ta) were obtained. The first-principle calculations were executed by CASTEP [14,26] and DMol3 [27,28], and the SHG coefficients were calculated using the optados [29] software by the classical anharmonic oscillator (AHO) model [3,12,30,31,32].

## 2. Materials and Methods

### 2.1. Model of BaMO(IO_3_)_5_

**Figure 1:**
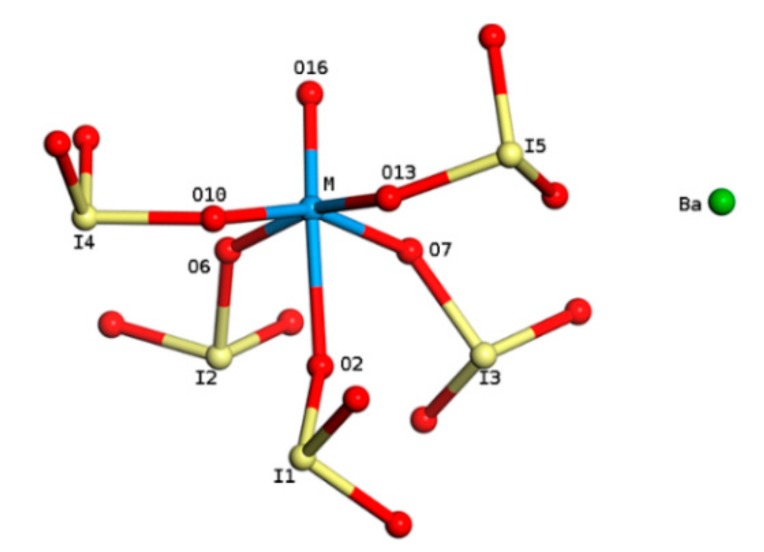
Atom structure of a BaMO(IO_3_)_5_ unit.

BaNbO(IO_3_)_5_ crystallizes in the non-centrosymmetric space group Cc (No. 9). In constructing the models of BaMO(IO_3_)_5_ (M = V, Ta), we firstly replaced the Nb atom in BaNbO(IO_3_)_5_ with V and Ta, respectively, and then carried out geometry optimizations for them. The atom structure of a BaMO(IO_3_)_5_ unit (molecular) is illustrated in Figure 1, in which the anionic group MO(IO_3_)_5_^2−^ shows a typical “zero-dimensional” structure: the M atom is in the center of a MO_6_ octahedron, and it links to five oxygen atoms in iodate groups and one terminal oxygen atom. This anionic group plays the main role in the SOJT effect thus the SHG response [18], and in affecting *E_g_* of the crystal, while the Ba^2+^ cation just acts as a spacer between the anions.

### 2.2. Properties Investigated

As the SHG response comes from the SOJT effect, and the SOJT effect can be reflected by the non-centrosymmetry of a crystal, a parameter, ∆*d*, defined in Equation (1), is estimated to represent the out-of-center distortion of the MO_6_ octahedron.

(1) $$\Delta d=\frac{\left(\left|{M-O}_{2}\right|-\left|{M-O}_{16}\right|\right)}{\left|\cos∠O_{2}{-M-O}_{16}\right|}+\frac{\left(\left|{M-O}_{10}\right|-\left|{M-O}_{7}\right|\right)}{\left|\cos∠O_{10}{-M-O}_{7}\right|}+\frac{\left(\left|{M-O}_{6}\right|-\left|{M-O}_{13}\right|\right)}{\left|\cos∠O_{6}{-M-O}_{13}\right|\;}\;$$

where M and O_x_ denote the transition metal and oxygen atom indexed in x respectively as shown in Figure 1; |M-O_x_| is the bond length, and ∠O_x_-M-O_x_ is the bond angle.

More fundamentally, the SOJT effect comes from the asymmetric of the electronic distribution. Therefore, the local dipole moments of the MO_6_ and IO_3_^−^ polyhedra in a MO(IO_3_)_5_^2−^ are calculated based on analyzing the Mulliken charges of each atom.

Quantitative SHG coefficients are estimated by the AHO model; the SHG tensor component *d_ij_* can be expressed by $d_{ij}=1/2\chi^{\left(2\right)}(\omega )$, where $\chi^{\left(2\right)}(\omega )$ is the second-order nonlinear susceptibility. Meanwhile, $\chi^{\left(2\right)}(\omega )$ is a function of the first-order susceptibility $\chi^{\left(1\right)}(\omega )$ [33],

(2) $$\chi^{\left(2\right)}(2\omega ,\omega ,\omega )=\frac{\varepsilon_{0}^{2}ma}{N^{2}e^{3}}\chi^{\left(1\right)}(2\omega ){\left[\chi^{\left(1\right)}(\omega )\right]}^{2}\;$$

where $\chi^{\left(1\right)}(\omega )$ is a function of the complex dielectric function *ε*(*ω*),

(3) $$\chi^{\left(1\right)}{\left(\omega \right)}_{ii}=\frac{\left[\varepsilon {\left(\omega \right)}_{ii}-1\right]}{4\pi }\;$$

In Equation (2), *m* is the electron mass; *e* is electron charge; *ε*_0_ is the permittivity of vacuum; *N* is the density number of molecules in a crystal [32]. *a*, which characterizes the nonlinear response, can be further written as $a=\omega_{0}^{2}/d$. In the equation above, *ω*_0_, determined by *E_g_*, is the electronic transition frequency in the molecule; and *d* is the lattice constant, which can be estimated by $d={\left(1/N\right)}^{1/3}$.

From Equations (2) and (3), it can be easily identified that the foremost important parameter in the AHO model is the complex dielectric function tensor,

(4) $$\left[\begin{array}{ccc} \varepsilon_{11} & \varepsilon_{12} & \varepsilon_{13}\\ \varepsilon_{21} & \varepsilon_{22} & \varepsilon_{23}\\ \varepsilon_{31} & \varepsilon_{32} & \varepsilon_{33} \end{array}\right]\;$$

where each element *ε*(*ω*) can be written as *ε*(*ω*) = *ε*_1_(*ω*) + *iε*_2_(*ω*). In CASTEP, the imaginary part of the dielectric constant is estimated by

(5) $$\varepsilon_{2}(q\to O_{u},h\omega )=\frac{2e^{2}\pi }{\Omega \varepsilon_{0}}{\sum_{k,v,c}\left|\langle \Psi_{k}^{c}|u\cdot r|\Psi_{k}^{v}\rangle \right|}^{2}\delta (E_{k}^{c}-E_{k}^{v}-E)\;$$

where *u* is the vector defining the polarization of the incident electric field. This expression is similar to Fermi’s Golden rule for time-dependent perturbations; therefore, *ε*_2_(*ω*) can be thought of as detailing the real transitions between occupied and unoccupied electronic states.

The real and imaginary parts of the dielectric constant can be linked by a Kramers-Kronig transform, by which the real part of the dielectric function, *ε*_1_(*ω*), can be obtained as

(6) $$\varepsilon_{1}(\omega )=1+\frac{4}{\pi }P\int_{0}^{\infty }dx\frac{x\varepsilon_{2}(x)}{x^{2}-\omega^{2}}\;$$

where *P* denotes the principal value of the integral.

We have used the optados code to process the CASTEP results to obtain the SHG tensor components. In addition, to keep in touch with the experiment measurements [34], the SHG tensor components at *λ* = 1064 nm, which correspond to a photon energy of *ω* = 1.165 eV, are obtained. With the restriction of Kleinman’s symmetry, only ten independent SHG tensor components remain.

Given the complex dielectric function, all other linear optical properties can be also calculated. Here, the absorption function, $Abs=\varepsilon_{2}\omega /(nc)$, vital in characterizing the transmission of light, and the birefringence $\Delta n=n_{e}-n_{o}$, imperative for phase-matching in practical applications, were calculated. Here *n_o_* and *n_e_* are the refractivities for the ordinary-ray and the extraordinary-ray, respectively, and they can be obtained by $n^{2}(\omega )=\varepsilon (\omega )$.

Band-gap is another important parameter of an infrared crystal, as it determines the LDT. Therefore, the band structures of each crystal were also investigated. To make clear which groups contribute to the SHG effect, the partial density of states (DOS) around Fermi level combined with wavefunction isosurfaces are also analyzed. Above all, as the BaVO(IO_3_)_5_ and BaTaO(IO_3_)_5_ are both hypothetical, their lattice vibration spectra and dynamics simulations were also calculated to verify their stability.

### 2.3. Computation Details

Calculations on the geometry structures, electronic and optical properties were all carried out using the total-energy code of CASTEP. In geometry optimization, the Perdew-Burke-Ernzerhof functional of solid (PBEsol) was used according to Ref. [35], as it has been tested to be the most appropriate to predict the unit cell of BaMO(IO_3_)_5_, with an error ratio less than 0.8%. The spin-polarized effect was considered and the convergence thresholds for energy change, maximum force, maximum stress, and maximum displacement were set as 5 × 10^−6^ eV/atom, 0.01 eV/Å, 0.02 GPa and 5 × 10^−4^ Å, respectively.

The single-point energy was calculated within the framework of nonlocal gradient-corrected approximations, i.e., the PBE functional [36]. The interactions between the ionic cores and the electrons were described by Norm-Conserving pseudopotential [37]. The following orbital electrons were treated as valence electrons: Ba-5s^2^5p^6^6s^2^, Ta-5d^3^6s^2^, V-3d^3^4s^2^, Nb-4d^4^5s^1^, I-5s^2^5p^5^ and O-2s^2^2p^4^. The number of plane waves included in the basis was determined by a cutoff energy of 830 eV. The numerical integration of the Brillouin zone was performed using a 4 × 4 × 2 Monkhorst-Pack k-point sampling for BaMO(IO_3_)_5_, which has been carefully tested to obtain enough accuracy. In addition, 373 empty bands (energy range of 20 eV) were used in our calculations, which are enough for optical properties. 

The band-gaps predicted by PBE functional are usually much smaller than the experimental data due to the discontinuity of XC energy. Fortunately, it can usually give the accurate shape of the band structures. Therefore, the scissor-corrected PBE method can be applied widely to research NLO materials even without any experimental data [13,38,39]. This actually provides a simple way to design new NLO materials and predict their optical properties. The hybrid functional HSE06 [40] can give a much accurate prediction of band structures, therefore, in our calculations, the value of the scissor operator is set to be the difference between HSE06 and PBE *E_g_*. An exact calculation for the band structure, as well as *E_g_*, is critical, as the imaginary part of the dielectric constant reveals the electronic transitions among bands and *E_g_* determines the LDT of a crystal.

The vibration spectra were calculated using the DMol3 code with the same functional, GGA-PBE; and k-point sampling was set to 3 × 3 × 1. The DFT semi-core pseudopotential was adopted for core treatment and the basis set was DNP.

The ab initio molecular dynamics simulations were performed by CASTEP based on the above optimized crystal. The same functional and pseudopotential as above were used, while only the Gamma point was selected in k-point sampling. The time step was set as 1.0 fs. For BaVO(IO_3_)_5_, dynamic simulation of 1.5 ps was carried out at 600 K; and for BaTaO(IO_3_)_5_, dynamic simulations of 2.0 ps were carried out at 600 K, 500 K and 450 K.

## 3. Results and Discussion

### 3.1. The Kinetic Stability and Thermodynamic Stability of BaMO(IO_3_)_5_ (M = V, Ta)

**Figure 2:**
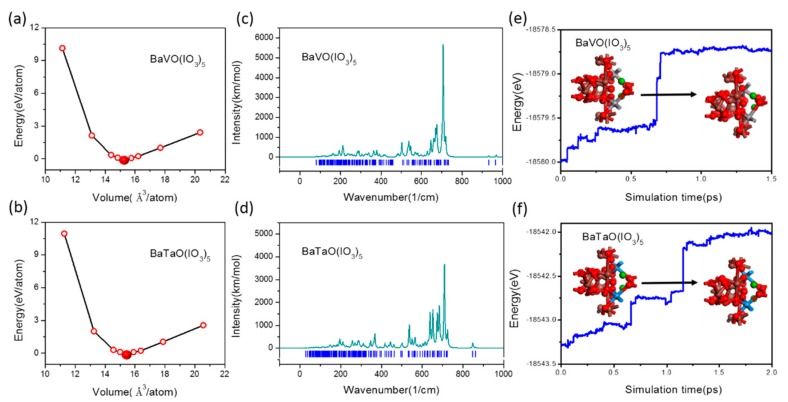
Evidence for the kinetic and thermodynamic stability of BaMO(IO_3_)_5_ (M = V, Ta). (**a**,**b**) The total energy per atom as a function of volume per atom for BaMO(IO_3_)_5_ (M = V, Ta), and (**c**,**d**) the lattice vibration spectra of them. (**e**,**f**) The energy evolution curves of the BaMO(IO_3_)_5_ (M = V, Ta) during the dynamics simulation.

As BaMO(IO_3_)_5_ (M = V, Ta) are hypothetical, before investigating their optical properties, we need to validate their kinetic stability in order to judge whether they are possible to be synthesized. For this purpose, we firstly plotted the energy curves versus the lattice volume for BaMO(IO_3_)_5_ (M = V, Ta), as given in Figure 2a,b. It can be seen that for both BaVO(IO_3_)_5_ and BaTaO(IO_3_)_5_, there is only one minimum point corresponding to their optimized structure in the wide range of volume modifications. This result indicates that they both have kinetically stable structures [41]. Then, we carried out lattice vibration analyses on the optimized structures of BaMO(IO_3_)_5_ (M = V, Ta) to further confirm their kinetic stability. Their obtained lattice vibration spectra are given in Figure 2c,d. Clearly, there is no imaginary frequency for both BaVO(IO_3_)_5_ and BaTaO(IO_3_)_5_, constituting more reliable evidence for their kinetic stability. Therefore, both BaVO(IO_3_)_5_ and BaTaO(IO_3_)_5_ are possible to be synthesized [42]. However, this evidence cannot guarantee that they are the most thermodynamically stable.

Therefore, we carried out ab initio molecular dynamics simulations to check the thermodynamic stability of these two compounds. The energy evolution curves of BaVO(IO_3_)_5_ and BaTaO(IO_3_)_5_ are shown in Figure 2e,f, respectively. For BaVO(IO_3_)_5_, the energy changes to fluctuate around a constant value after 0.7 ps, which means the system reaches an equilibrium state. In addition, from the inset in Figure 2e, it can be seen that the unit cell of BaVO(IO_3_)_5_ keeps its intrinsic structure after a dynamics process at 600 K, which indicates that BaVO(IO_3_)_5_ is the most thermodynamically stable. In addition, we can find that all the atoms vibrate around their balance positions during the dynamics simulations, as shown in the Supplementary Materials. Similar results were obtained for BaTaO(IO_3_)_5_, except for that it is the most thermodynamically stable only under 450 K. The simulation results at 600 K and 500 K are given in the Supplementary Materials.

### 3.2. Reliability of Our Predictions on the NLO Properties

To ensure the reliability and precision of our calculated results is the premise for the significance of our predictions on the NLO properties of BaMO(IO_3_)_5_ (M = V, Ta). To this end, we calculated many properties of BaNbO(IO_3_)_5_ and compared them with reported calculated and experimental results, as given in Table 1.

At first, it can be seen that the lattice parameters we calculated are nearly the same as the experimental measurements, with a maximum error of 0.7%. This ensures the reliability of our predictions on the geometrical structures of BaMO(IO_3_)_5_ (M = V, Ta), which is the basis for studying the electronic properties. Meanwhile, the accurate lattice parameters also ensure the accuracy for the calculation of Δ*d* (Equation (1)) and the SHG coefficients which need an input parameter *N* (Equation (2)). Then, we calculated the band-gap of BaNbO(IO_3_)_5_ using the hybrid functional HSE06, 3.55 eV, which is very similar to (slightly smaller than) the experimental value, 3.64 eV. This result is more accurate than the reported calculated value, which is obtained using the PBE functional with lattice parameters from experimental measurements. This result confirms the reliability of using HSE06 functional in calculating *E_g_* of BaMO(IO_3_)_5_. Combined with the accuracy of the PBE in predicting the shape of band structures, such a result can ensure the reliability of our predictions on both the SHG response and *E_g_* of BaMO(IO_3_)_5_ (M = V, Ta). Dielectric function is the foremost essential parameter in calculating the SHG coefficients. The veracity of our calculations on dielectric function can be verified by the fact that the static dielectric constant *ε*_(0)_ we calculated is very comparable to the reported calculated results. This ensures the accuracy of our predictions on the SHG response of BaMO(IO_3_)_5_ (M = V, Ta) and on the birefringence Δ*n*, which is a determining parameter for phase-matching conditions.

As a verification, the value of our calculated SHG coefficient, *d*_11_, at 1064 nm is very close to but more precise than the reported calculated result compared with the experimental value. This enhancement comes from the fact that we did not use the experimental lattice parameters directly [12], but carried out geometry optimization before calculating the electronic properties. Additionally, the birefringence Δ*n* we obtained is also close to the reported calculated value. In a word, the calculation scheme and settings we used can warrant the legitimacy of our predictions on the NLO properties of BaMO(IO_3_)_5_ (M = V, Ta).

### 3.3. NLO Properties of BaVO(IO_3_)_5_ and BaTaO(IO_3_)_5_

The SHG coefficient and LDT are the two main indices in estimating whether a crystal is practical for NLO conversion. SHG coefficient determines the nonlinear frequency conversion efficiency of a crystal, and LDT determines the maximum optical power density that the crystal can withstand. Considering the spatial symmetry and the Kleinman’s full permutation symmetry condition for a lossless nonlinear medium, crystal class with *m* point group has six independent SHG tensor components (*d*_11_, *d*_12_, *d*_13_, *d*_15_, *d*_24_ and *d*_33_), which are used to characterize the SHG coefficient. In addition, as LDT is generally in proportion to *E_g_* of a crystal, here, we use *E_g_* to represent LDT.

The frequency-dependent SHG tensor components of BaMO(IO_3_)_5_ at a wavelength of 1064 nm (1.165 eV) are listed in Table 2. It can be seen that BaVO(IO_3_)_5_ has pretty large SHG coefficients: the maximum component *d*_12_ reaches 23.42 × 10^−9^ esu, which is about 25.5% larger compared with that of BaNbO(IO_3_)_5_, 18.66 × 10^−9^ esu. Such a great SHG coefficient means that BaVO(IO_3_)_5_ is likely to provide a high nonlinear frequency conversion efficiency. For BaTaO(IO_3_)_5_, its biggest component of SHG coefficient is slightly smaller than that of BaNbO(IO_3_)_5_, but it is also quite large, *d*_11_ = 17.02 × 10^−9^ esu, which means that BaTaO(IO_3_)_5_ can also provide high NLO conversion efficiency.

**Figure 3:**
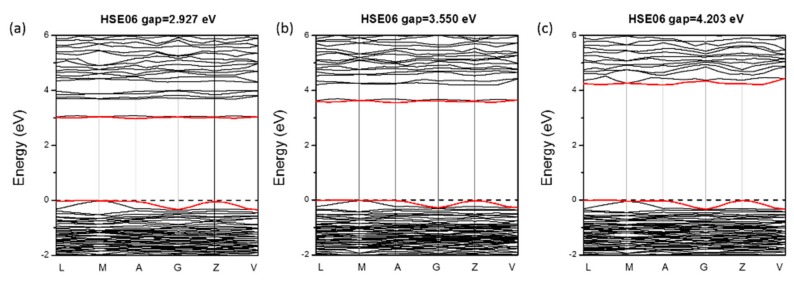
The band structures of BaMO(IO_3_)_5_. (**a**) BaVO(IO_3_)_5_; (**b**) BaNbO(IO_3_)_5_; (**c**) BaTaO(IO_3_)_5_. The Fermi level is set at 0 eV; the red lines denote the VBM and CBM.

LDT has become the major limitation for the practical application of many new materials [9,13,15]. A typical lower limit of LDT in judging whether one crystal satisfies the practical requirement is 100 MW/cm^2^ which corresponds to *E_g_* > 3.0 eV. From the electronic band structures in Figure 3, it can be seen that *E_g_* of BaVO(IO_3_)_5_ is 2.927 eV, which is very close to 3.0 eV. Considering that *E_g_* is always underestimated by PBE, even if using the HSE06 functional [43] (e.g., as in Table 1, the calculated *E_g_* of BaNbO(IO_3_)_5_ is 0.09 eV smaller than the experimental value, 3.55 vs. 3.64 eV), the real *E_g_* of BaVO(IO_3_)_5_ is very likely to be greater than 3.0 eV, which satisfies the practical requirement. For BaTaO(IO_3_)_5_, it can be observed from Figure 3c that its *E_g_* reaches 4.203 eV. If we take the underestimation of PBE method into account, its real *E_g_* may be somewhat larger. Such a great *E_g_* can effectively suppress the two-photon and multi-photon absorptions; therefore, BaTaO(IO_3_)_5_ is possible to have a much larger LDT than BaVO(IO_3_)_5_.

Besides SHG and LDT, an infrared NLO crystal should also have an appropriate birefringence Δ*n*, whose value should be in the range of 0.03–0.1 to satisfy the phase-matching condition. The refractive index *n* and birefringence Δ*n* of BaMO(IO_3_)_5_ at 1064 nm are given in Table 3. It is shown that the birefringence of BaVO(IO_3_)_5_ is 0.04, which completely meets the requirement. Meanwhile, the birefringence of BaTaO(IO_3_)_5_ is only 0.02, which seems to fail to satisfy the phase-matching condition. Nevertheless, it should be noted here that Δ*n* of BaMO(IO_3_)_5_ may be underestimated in our calculations. This assumption is based on the fact that the calculated Δ*n* of BaNbO(IO_3_)_5_ is also only 0.02 (Table 3), while still being able to support perfect phase-matching in practice [12]. In other words, Δ*n* of BaTaO(IO_3_)_5_ may also satisfy the phase-matching condition, but this requires further confirmation. At the very least, even if a bare BaTaO(IO_3_)_5_ crystal cannot realize phase-matching, its *Δn* can also be manipulated by pressure engineering [7].

**Figure 4:**
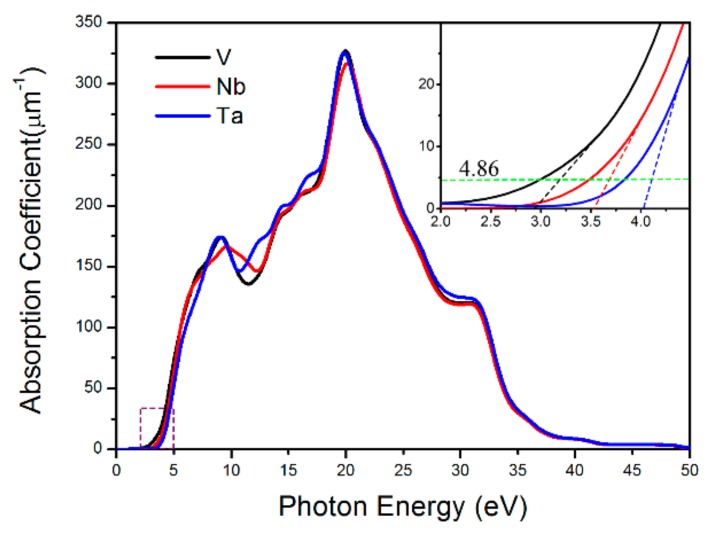
The absorption spectra of BaMO(IO_3_)_5_.

In addition to the large SHG coefficient, large LDT, and appropriate Δ*n*, an excellent infrared NLO crystal should also have a wide transparent wavelength region with low optical absorption. The absorption spectra of BaMO(IO_3_)_5_ are given in Figure 4. In the ultraviolet region (3.0~38.4 eV), BaMO(IO_3_)_5_ shows large absorption coefficients, which is in agreement with experimental results [12], while in the visible-infrared region (<3.0 eV), the absorption coefficients decrease sharply and become very small. These optical absorption properties indicate that these three crystals are all suitable for infrared NLO materials. From the inset in Figure 4, it can be also recognized that the transparent wavelength region of BaMO(IO_3_)_5_ widens gradually with M varies from V to Nb then to Ta, and this is consistent with the gradually increased *E_g_*.

In summary, BaVO(IO_3_)_5_ has larger SHG coefficients than BaNbO(IO_3_)_5_, its *E_g_* satisfies the basic requirement *E_g_* > 3.0 eV, its Δ*n* is appropriate for an infrared NLO crystal, and its light absorption coefficient is pretty low in the wide visible-infrared region. The SHG coefficient and band-gap of BaVO(IO_3_)_5_ are close to the calculated results of NaVO_2_(IO_3_)_2_(H_2_O). BaTaO(IO_3_)_5_ also has quite a large SHG coefficient (only slightly decreased compared with BaNbO(IO_3_)_5_); moreover, its *E_g_* is significantly greater than that of BaNbO(IO_3_)_5_, which indicates a much higher LDT. Additionally, BaTaO(IO_3_)_5_ has a wider transparent wavelength region than BaNbO(IO_3_)_5_; and its birefringence Δ*n* can basically meet the requirement. BaVO(IO_3_)_5_ and BaTaO(IO_3_)_5_ both are expected to be excellent infrared NLO materials.

### 3.4. Effect of Element Substitution on the NLO Performance

The different SHG performance of BaMO(IO_3_)_5_ (M = V, Nb and Ta) originally comes from the SOJT effect. From the aspect of geometry configuration, the SOJT effects can be represented by *∆d* as given in Equation (1), which describes the out-of-center distortion in MO(IO_3_)_5_^2−^ octahedron. The calculated ∆*d* of the VO_6_, NbO_6_, and TaO_6_ octahedrons are 1.004, 0.605 and 0.663, respectively. The bond lengths and bond angles used in the calculation of ∆*d* are given in Table 4. This sequence can be reasonably explained by the increasing ionic radiuses of the three congener elements, *R_V5+_ =* 5.3 Å, *R_Nb5+_ =* 6.4 Å, and *R_Ta5+_ =* 6.4 Å [44]. V^5+^ has the smallest ionic radius which results in the strongest SOJT effect. Meanwhile, Nb^5+^ and Ta^5+^, have nearly the same, but much greater radii than V^5+^; therefore, they lead to slightly weaker SOJT effects than V^5+^. This explanation is generally consistent with the reduced SHG coefficients of BaMO(IO_3_)_5_ (M = V, Nb and Ta), 23.42, 18.66 and 17.02 × 10^−9^ esu.

**Figure 5:**
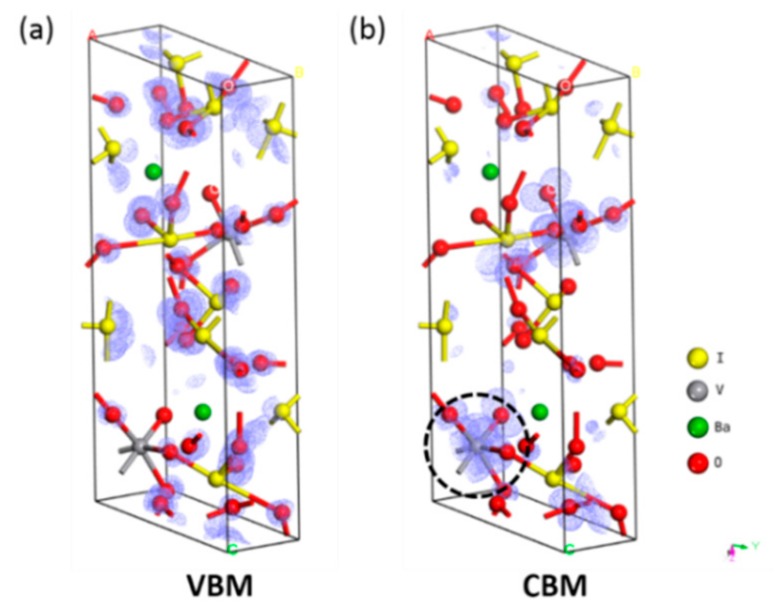
The wavefunction isosurface plots at (**a**) the VBM and (**b**) the CBM of BaVO(IO_3_)_5_.

More fundamentally, the SHG response comes from the asymmetric electronic distribution in BaMO(IO_3_)_5_. Therefore, the local dipole moment should be a better indicator of the SOJT effect. Nonetheless, before calculating the local dipole moments in BaMO(IO_3_)_5_, we firstly demonstrated that only the IO_3_^−^ and V^5+^ play the important role in the large SHG response of BaMO(IO_3_)_5_. We mapped the wavefunction isosurfaces for the VBM and CBM of BaVO(IO_3_)_5_ in Figure 5, as the optical response mainly originates from the electron transitions around the band-gap. It can be seen that in the VBM, a large number of electrons concentrating on the IO_3_^−^ units but no electron distributes around V^5+^; in the CBM, some electrons transfer from the IO_3_^−^ units to V^5+^, as denoted by the black dash circle in Figure 5b. Such an electron distribution is very important for the generation of macroscopic second order polarization [9], which indicates that only the IO_3_^−^ units and V^5+^ play an important role in producing the SHG response. These results are in accordance with the anionic group theory and the research by Lei [13] for infrared NLO crystals.

According to the above analyses, only local dipole moments of the MO_6_ and IO_3_^−^ polyhedrons in the anionic group were calculated. The calculation method we used is the same as the reported one [45,46,47,48,49,50], but the charges of atoms were Mulliken charges which may be more appropriate than those calculated by the bond-valence theory. The detailed local dipole moments of each MO_6_ and IO_3_ groups are given in Table 5. It can be seen that the net dipole moments (NDM) for one anionic group in BaVO(IO_3_)_5_, BaNbO(IO_3_)_5_ and BaTaO(IO_3_)_5_ are 89.93, 83.12 and 80.84, respectively. This consists with the general rule of the SOJT effect for d^0^ metal: V^5+^ > Nb^5+^ > Ta^5+^ [8], and agrees very well (better than ∆*d*) with it, and thus can accurately explain the sequence of the SHG coefficients of the three crystals.

**Figure 6:**
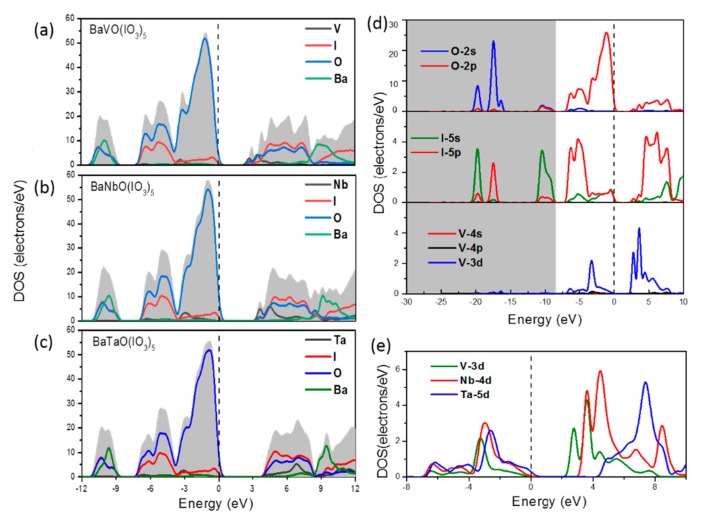
PDOS projected to the constituent atoms of (**a**) BaVO(IO_3_)_5_; (**b**) BaNbO(IO_3_)_5_; (**c**) BaTaO(IO_3_)_5_; (**d**) PDOS of the O-, I-, and V-orbitals in BaVO(IO_3_)_5_; and (**e**) PDOS of the M-d orbitals.

The band-gap of a BaMO(IO_3_)_5_ crystal is significantly determined by the electronegativity of the M atom. As the electronegativity decreases from V to Nb and then to Ta, the band-gaps of BaVO(IO_3_)_5_, BaNbO(IO_3_)_5_, and BaTaO(IO_3_)_5_ are expected to increase in sequence. This roughly explains the results of *E_g_* in Figure 2, whose values are 2.927 eV, 3.55 eV and 4.203 eV, respectively for BaVO(IO_3_)_5_, BaNbO(IO_3_)_5_ and BaTaO(IO_3_)_5_. More detailed effects of congener substitution on *E_g_* should be analyzed by combining the electronic band structures and DOS. The band structures are given in Figure 2 and some PDOS figures used to analyze the contribution of different atomic orbitals are given in Figure 6a–e.

From Figure 6a–c, it can be seen that the PDOS of Ba distributes far away from the Fermi level, which is mainly located in regions of *E_ele_* < −9 eV and *E_ele_* > 8 eV. This suggests that the Ba atom takes almost no part in the process of light absorption, which also confirms the anionic group theory. Next, the contributions of different atoms in the anionic group on the bandstructure will be analyzed. Due to space limitations, only the PDOS of BaVO(IO_3_)_5_ is given here in Figure 6d. We can see that the valence band (from −7 eV to Fermi level) is mainly composed of O-2p, I-5s, I-5p, and V-3d states, while the conductive band mainly consists of V-3d, I-5p, and O-2p states. Hence, the transition metal M makes contributions to both the valance and conductive bands. To compare the different contributions of these three transition metals, the PDOS of the V-3d, Nb-4d, and Ta-5d orbitals are plotted in Figure 6e. In the regions below the Fermi energy level, their shapes are nearly the same, which demonstrates that they contribute similarly on the valence band of a BaMO(IO_3_)_5_ crystal. The only difference is that the position of their peaks increases with a sequence V-3d, Nb-4d, and Ta-5d, which reflects the decreased electronegativity. During the time, in the conduction band regions, the PDOS of V-3d, Nb-4d and Ta-5d in the conduction band regions shows some discrepancies; the shapes for V-3d and Nb-4d are similar, albeit that of Ta-5d is not the same. This different situation of the Ta-5d orbital can be analyzed by analyzing its hybrid situation with other orbitals.

From Figure 6a,b, it can be seen that:

(1) There is an obvious sharp peak at the bottom of the conduction band, and from Figure 6d,e we can see that this peak results from the contributions of V-3d and Nb-4d;
(2) In the conduction band region, the DOS of I and O are very comparable, which exhibits the full hybrid interaction in IO_3_^−^; at the same time, their energy range (I and O) is intertwined with that of the transition metal V and Nb.

These results suggest that some electrons in the M-O bond in BaVO(IO_3_)_5_ and BaNbO(IO_3_)_5_ are localized around the M atom under excited states, and therefore, the M-d orbital in the conduction band shows weak hybridization with the O-2p, I-5s, and I-5p orbitals. In Figure 6c (for BaTaO(IO_3_)_5_), the sharp peak vanishes, and it can be seen that the DOS of I and O is distinctly different from that in Figure 6a,b, which has a rapidly increasing conduction band edge and an energy range overlapping with the DOS of Ta. This means that the electrons on the Ta-5d orbital are delocalized and Ta-5d has stronger hybridization with the O-2p, I-5s, and I-5p orbitals. This is consistent with the weakest electronegativity of Ta. Combined with the SHG results, it can be concluded that although Ta contributes similarly to Nb to the SHG response of the BaMO(IO_3_)_5_ crystal, its excited d-orbital is delocalized and has stronger hybrid interaction with IO_3_^−^ than Nb-4d, which lifts the energy of the conductive band, thus resulting in a larger *E_g_*.

**Figure 7:**
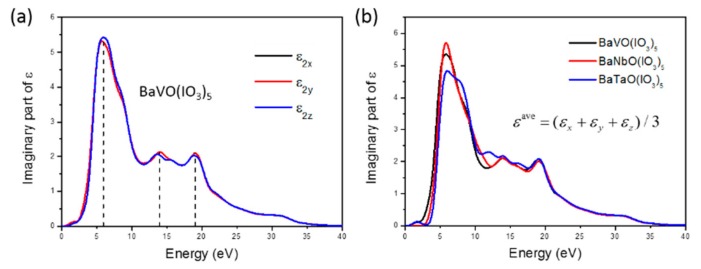
(**a**) Imaginary part *ε*_2_(*ω*) of the frequency-dependent dielectric function of BaVO(IO_3_)_5_ along different dielectric axes. (**b**) Averaged *ε*_2_(*ω*) of BaMO(IO_3_)_5_ (M = V, Nb and Ta).

The light absorption properties of the BaMO(IO_3_)_5_ crystals can also be explained by the band structures. In Figure 2, it can be found that BaVO(IO_3_)_5_, BaNbO(IO_3_)_5_ and BaTaO(IO_3_)_5_ are all direct-gap semiconductors based on the fact that both VBM and CBM locate at the middle point of the LM line. Direct-gap means a fast-decaying optical absorption coefficient near the absorption edge, as shown in Figure 4, which is favorable for an infrared NLO crystal. More directly, the light absorption ability of a material can be revealed by the imaginary part *ε*_2_(*ω*) of the dielectric function *ε*(*ω*), as given in Figure 7a. Here, only the results of *ε*_2_(*ω*) for BaVO(IO_3_)_5_ are given due to space limitations. At first, it can be seen that *ε*_2_(*ω*) of BaVO(IO_3_)_5_ shows a slight anisotropy along different dielectric axes. This is the original reason of the birefringence Δ*n*, which can be attributed to the fact that the lone pairs in IO_3_^−^ units are arranged in parallel in BaVO(IO_3_)_5_. Then, the averaged *ε*_2_(*ω*) of BaVO(IO_3_)_5_ are plotted in Figure 7b, where *ε*_2_(*ω*) of all three crystals are pretty low when the photon energy is less than 3.5 eV. This indicates that all three BaMO(IO_3_)_5_ have very low light absorption in the visible and infrared wavebands. 

From Figure 7a, it can also be seen that there are many peaks in the curves of the dielectric function, which reveals the electronic transitions between different bands. Of these, the first peak of BaMO(IO_3_)_5_, whose value is about 5.8 eV, corresponds to the absorption of direct transition at absorption edge. Combined with Figure 6a,d, we can find that this peak is mainly contributed by the transition from the VBM to the excited O-2p, I-5p, and M-d (V-3d, Nb-4d, Ta-5d) orbitals. Additionally, the values of peak-1 for BaVO(IO_3_)_5_, BaVO(IO_3_)_5_, and BaVO(IO_3_)_5_ show a few disparities, which are 5.79 eV, 5.82 eV and 5.91 eV, respectively. This variation tendency of theirs is in keeping with that of *E_g_* of the three crystals. In addition, it can also be seen that peak-1 of BaTaO(IO_3_)_5_ is broadened, which can be explained by the stronger hybrid interactions between the Ta-5d and the I-5p, O-2p orbitals.

In summary, with variation of the M in BaMO(IO_3_)_5_ to V, Nb and Ta, the SHG coefficient of BaMO(IO_3_)_5_ decreases, while the band-gap (*E_g_*) of BaMO(IO_3_)_5_ increases. This is coherent with the rule that a strong SHG effect and a large band-gap are a pair of trade-off indices [9,13,38]. This is because the SOJT effect intrinsically derives from the interaction between the VBM and CBM orbitals: if *E_g_* of the system containing the SOJT anions is too large, the interaction between VBM and CBM would be very small, which would decrease the SOJT effect. Therefore, it is necessary to strike a balance between a large band-gap and a large SHG response in the design of new infrared NLO crystals.

## 4. Conclusions

In this work, two promising NLO crystals, BaVO(IO_3_)_5_ and BaTaO(IO_3_)_5_, are designed, and the main optical indices for their practical application are predicted based on first principle calculations. At first, we demonstrated that they have no imaginary frequency vibrations, which verifies that they are kinetically stable, and thus are possible to be synthesized. The ab initio molecular dynamics simulations were performed at high temperatures to testify their thermodynamic stabilities. Then, the reliability of our calculations is validated by comparing our predicted *E_g_* and SHG coefficients with the measured values for BaNbO(IO_3_)_5_. Next, the NLO properties of BaMO(IO_3_)_5_ (M = V, Ta) are investigated, where the SHG coefficients are calculated by classical AHO model. Finally, the effects of substituting Nb with V and Ta on the NLO performance of BaMO(IO_3_)_5_ are analyzed in detail. 

It is demonstrated that BaVO(IO_3_)_5_ has a 25.5% enlarged SHG coefficient compared with BaNbO(IO_3_)_5_, a big *E_g_* that satisfies the general criterion of practical application (*E_g_* > 3.0 eV), an appropriate birefringence meeting the requirement of phase matching, and a pretty low light absorption coefficient within a wide visible-infrared region. With regard to BaTaO(IO_3_)_5_, it also has quite a large SHG coefficient (slightly smaller than that of BaNbO(IO_3_)_5_). Furthermore, its *E_g_* is significantly greater than that of BaNbO(IO_3_)_5_ (4.203 vs. 3.550 eV), which indicates an improved LDT; meanwhile, it shows wider transparent wavelength region than BaNbO(IO_3_)_5_. Last but not least, its birefringence also meets the practical applications. In summary, BaVO(IO_3_)_5_ and BaTaO(IO_3_)_5_ both are expected to be excellent infrared NLO materials.

The different SHG responses of BaMO(IO_3_)_5_ (M = V, Nb and Ta) come from the distinct SOJT effects brought along by the V, Nb, and Ta atoms. In terms of geometry configuration, the SOJT effect can be inferred by ∆*d* of the VO_6_, NbO_6_, and TaO_6_ octahedrons, which are 1.004, 0.605 and 0.663, respectively; from a more fundamental point of view, the SOJT effect can be characterized by the local dipole moment of the BaVO(IO_3_)_5_, BaNbO(IO_3_)_5_, and BaTaO(IO_3_)_5_ units, which are 89.93, 83.12 and 80.84, respectively. Both the sequences of ∆*d* and the local dipole moment agree well with that of the SHG coefficients, and this explains the effect of congener element substitution on the SHG response on BaMO(IO_3_)_5_.

The gradually increasing *E_g_* of BaVO(IO_3_)_5_, BaNbO(IO_3_)_5_ and BaTaO(IO_3_)_5_ can be reasonably explained by the fact that the electronegativity of V, Nb, and Ta decreases in sequence. Such decreasing electronegativity results in the degree of hybridization between the M-d orbital and the I- and O-orbitals in the IO_3_^−^ group increasing, thus in gradually raising the energy of the conductive band. In particular, the Ta-d orbital hybridizes strongly with the O-2p, I-5s, and I-5p orbitals. Therefore, BaTaO(IO_3_)_5_ shows a significantly increased *E_g_*. 

The good light absorption performance can be attributed to the fact that BaMO(IO_3_)_5_ are all direct-gap crystals, which will result in fast-decaying optical absorption near the absorption edge. It is also found that the imaginary part of the dielectric function for BaMO(IO_3_)_5_ (M = V, Nb and Ta) has very small values when the photon energy is less than 3.5 eV, which justifies their very low light absorption coefficient over a wide visible and infrared waveband. The birefringence Δ*n* of BaMO(IO_3_)_5_ can be derived and thus interpreted by the anisotropy of their dielectric function along different dielectric axes. In addition, the effect of Ba on the NLO performance of BaMO(IO_3_)_5_ can be clearly excluded by the fact that its PDOS distributes far away from the Fermi level, which confirms the anionic group theory. We hope the discoveries and insights in this work are helpful for exploring new infrared NLO materials.

## Figures and Tables

**Table 1:** The lattice parameters, electronic and optical properties of BaNbO(IO_3_)_5_.

BaNbO(IO_3_)_5_		Our Results	Reference	
Lattice Parameter	*a* (Å)	7.95	–	7.93^exp^
	*b* (Å)	7.95	–	7.93^exp^
	*c* (Å)	23.90	–	24.08^exp^
	*α* (°)	136.09	–	136.46^exp^
	*β* (°)	136.09	–	136.46^exp^
	*γ* (°)	56.51	–	56.51^exp^
*E_g_* (eV)		3.55	2.55^cal^	3.64^exp^
*ε*_(0)_		4.51	4.50^cal^	–
∆*n*		0.02	0.03^cal^	–
*d*_11_ at 1064 nm (×10^−9^ esu)		18.66	19.80^cal^	15.40^exp^

Note: the calculated and experimental results both come from Ref. [12].

**Table 2:** The SHG tensor components of BaMO(IO_3_)_5_ (M = V, Nb and Ta) at 1064 nm.

SHG (×10^−9^ esu)	*d*_11_	*d*_12_	*d*_13_	*d*_15_	*d*_24_	*d*_33_
BaVO(IO_3_)_5_	23.24	23.42	22.05	22.64	23.03	21.23
BaNbO(IO_3_)_5_	18.66	18.39	17.97	18.31	18.18	17.60
BaTaO(IO_3_)_5_	17.02	16.58	16.26	16.64	16.19	15.85

**Table 3:** Refractive index *n* and birefringence Δ*n* of BaMO(IO_3_)_5_.

**Crystals**	***n_x_***	***n_y_***	***n_z_***	**∆** ***n***
BaVO(IO_3_)_5_	2.22	2.22	2.26	0.04
BaNbO(IO_3_)_5_	2.15	2.15	2.17	0.02
BaTaO(IO_3_)_5_	2.12	2.12	2.14	0.02

**Table 4:** Bond lengths, bond angles, and Δ*d* in BaMO(IO_3_)_5_ (M = V, Nb and Ta).

Parameters		BaVO(IO_3_)_5_	BaNbO(IO_3_)_5_	BaTaO(IO_3_)_5_
Bond lengths (Å)	M-O_16_	1.653	1.855	1.754
	M-O_6_	1.936	2.093	1.958
	M-O_7_	1.866	2.063	1.924
	M-O_10_	1.981	2.106	1.954
	M-O_13_	1.891	2.069	1.924
	M-O_2_	2.470	2.381	2.342
	O-I_1_	1.833	1.844	1.840
Bond angles (°)	O_16_-M-O_2_	169.52	170.58	170.98
	O_10_-M-O_7_	156.25	156.44	156.33
	O_6_-M-O_13_	159.25	165.49	165.42
Δ*d*		1.004	0.605	0.663

**Table 5:** Dipole moments for the IO_3_, VO_6_ polyhedra and the NDM for one anionic group.

Groups	Dipole Moment (D)											
	X-Component			Y-Component			Z-Component			Total Magnitude		
	M = V	M = Nb	M = Ta	M = V	M = Nb	M = Ta	M = V	M = Nb	M = Ta	M = V	M = Nb	M = Ta
I(1)O_3_	0.28	−0.34	−0.56	7.50	7.36	7.40	−11.11	−11.35	−11.31	13.40	13.53	13.53
I(2)O_3_	−1.46	−0.62	−1.60	−8.48	−8.39	−8.05	−10.57	−10.68	−10.67	13.63	13.60	13.46
I(3)O_3_	8.36	7.77	7.38	−6.95	−6.70	−7.51	−8.14	−8.51	−8.15	13.59	13.33	13.32
I(4)O_3_	−7.17	−6.50	−6.69	1.59	2.03	2.08	−10.60	−11.07	−10.98	12.90	13.00	13.02
I(5)O_3_	7.92	7.53	7.74	−9.82	−9.94	−9.92	−3.84	−3.73	−3.98	13.19	13.01	13.19
MO_6_	7.32	6.37	5.92	7.73	6.44	5.79	20.65	13.98	11.68	23.23	16.66	14.32
NDM	15.26	14.21	12.19	−8.43	−9.20	−10.20	−23.62	−31.37	−33.41	89.93	83.12	80.84

## Supplementary Materials

The following are available online at http://www.mdpi.com/1996-1944/11/10/1809/s1, Figure S1: The energy evolution curves of the BaTaO(IO_3_)_5_ at 600 K during the dynamics simulation; Figure S2: The energy evolution curves of the BaTaO(IO_3_)_5_ at 500 K during the dynamics simulation; Video S1: The trajectories of the dynamics simulation.
