# Supplementary material for: Excellent Infrared Nonlinear Optical Crystals BaMO(IO3)5 (M = V, Ta) Predicted by First Principle Calculations
Source: Materials (Basel). 2018 Sep 24;11(10):1809. doi: 10.3390/ma11101809 (PMC6213473; doi:10.3390/ma11101809)
Supplement: Supplementary file 1 [file materials-11-01809-s001.pdf]

materials

*Supporting information*

# Excellent Infrared Nonlinear Optical Crystals $BaMO(IO_3)_5$(M = V, Ta) Predicted by First Principle Calculations

**Yingfeng Li [1,*,†], Mengqi Cui [1,2,†], Hejin Yan [1], Yang-Xin Yu [2,3,*], Meicheng Li [1], Xiang Li [1], Lihua Chu [1], Bing Jiang [1] and Mingde Qin [4]**

1 State Key Laboratory of Alternate Electrical Power System with Renewable Energy Sources, School of Renewable Energy, North China Electric Power University, Beijing 102206, China; cmqroy@126.com (M.C.); li-bluesky@hotmail.com (H.Y.); mcli@ncepu.edu.cn (M.L.); 13051314181@163.com (X.L.); 51102229@ncepu.edu.cn (L.C.); mucaoshan@163.com (B.J.)

2 Laboratory of Chemical Engineering Thermodynamics, Department of Chemical Engineering, Tsinghua University, Beijing 100084, China

3 State Key Laboratory of Chemical Engineering, Department of Chemical Engineering, Tsinghua University, Beijing 100084, China

4 Program of Materials Science and Engineering, University of California, San Diego, CA 92093, USA; mingdeqin2@gmail.com

* Correspondence: liyingfeng@ncepu.edu.cn (Y.L.); yangxyu@mail.tsinghua.edu.cn (Y.Y.); Tel.: +86-10-61772332 (Y.L.); +86-10-62782558 (Y.Y.)

† Contributed equally in this work

Received: 09 August 2018; Accepted: 18 September 2018; Published: date

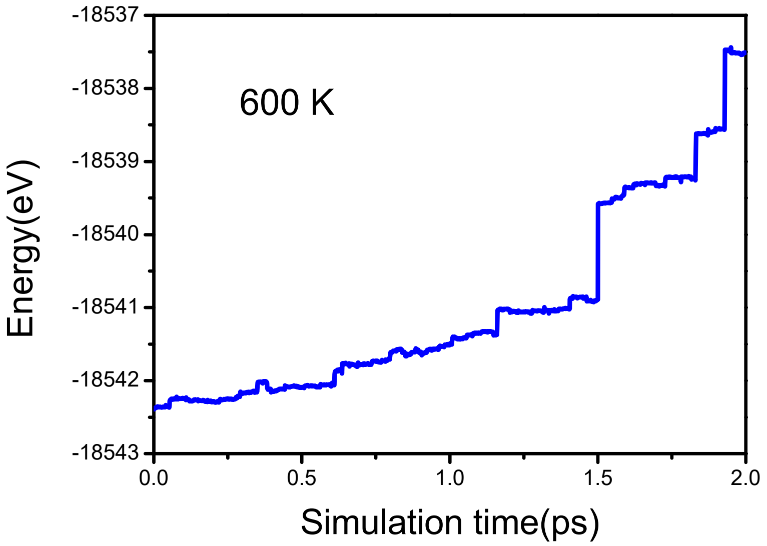


**Figure S1.** The energy evolution curves of the $BaTaO(IO_3)_5$ at 600 K during the dynamics simulation.

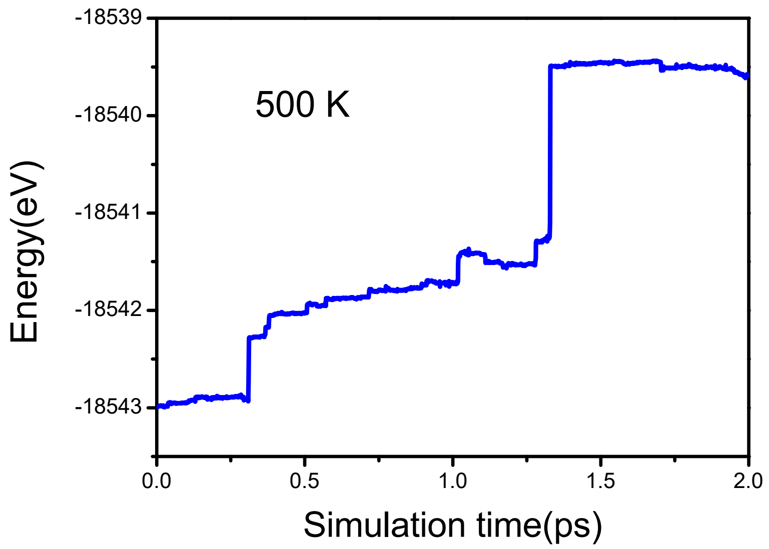


**Figure** S**2.** The energy evolution curves of the $BaTaO(IO_3)_5$ at 500 K during the dynamics simulation.

© 2018 by the authors. Submitted for possible open access publication under the terms and conditions of the Creative Commons Attribution (CC BY) license (http://creativecommons.org/licenses/by/4.0/).
